# The Effect of Auricular Acupressure on Pruritus, Xerostomia, and Fatigue in Patients Undergoing Hemodialysis: A Clinical Trial in Southeastern Iran

**DOI:** 10.1155/ijne/9943333

**Published:** 2026-08-19

**Authors:** Ehsan Behjat Jabari, Mansooreh Azizzadeh Forouzi, Batool Tirgari, Yunes Jahani, Tori Canillas Dufau, Mohammad Setayesh

**Affiliations:** ^1^ Physiology Research Center, Institute of Neuropharmacology, Kerman University of Medical Sciences, Kerman, Iran, kmu.ac.ir; ^2^ Neuroscience Research Center, Institute of Neuropharmacology, Kerman University of Medical Sciences, Kerman, Iran, kmu.ac.ir; ^3^ Nursing Research Center, Kerman University of Medical Sciences, Kerman, Iran, kmu.ac.ir; ^4^ Modeling in Health Research Center, Institute for Futures Studies in Health, Kerman University of Medical Sciences, Kerman, Iran, kmu.ac.ir; ^5^ Department of Nursing, Mount Saint Mary’s University, Los Angeles, USA; ^6^ Herbal and Traditional Medicines Research Center, Kerman University of Medical Sciences, Kerman, Iran, kmu.ac.ir

**Keywords:** auricular acupressure, fatigue, hemodialysis, pruritus, xerostomia

## Abstract

**Background:**

Itching, dry mouth, and fatigue as common problems in hemodialysis patients affect their quality of life. Auricular acupressure is a traditional Chinese medicine method that elicits reactions such as heat, numbness, expansion, pain, and other forms of stimulation to achieve therapeutic effects. This study aimed to assess the impact of auricular acupressure on alleviating pruritus, xerostomia, and fatigue in patients undergoing hemodialysis in southeastern Iran.

**Method:**

This clinical trial study was conducted in Shafa, Samen al‐Hojaj, and Javad Al‐Aemeh Hospitals. The sample size consisted of 90 individuals who were randomly assigned to either the control or intervention group using a two‐stage randomization method (first separated by odd/even dialysis days to prevent contamination, then clusters randomly allocated to groups). The intervention group received auricular acupressure treatment for a duration of 4 weeks, while the control group received routine care. Both groups completed clinical questionnaires, including the Visual Analog Scale (VAS), the Summated Xerostomia Inventory (SXI), and the Fatigue Severity Scale (FSS), before and after the intervention. The collected data were analyzed using SPSS26, employing both parametric and nonparametric tests, with a significance level set at 0.05.

**Results:**

The study findings indicated that auricular acupressure had a significant impact on the patients in the intervention group. The pruritus score decreased from 6.11 ± 2.32 to 3.18 ± 2.74, a reduction of 2.93 points (*p* < 0.001). The xerostomia score decreased from 19.64 ± 3.46 to 15.62 ± 5.29, a decrease of 4.02 points (*p* < 0.001). Additionally, the fatigue score decreased from 51.73 ± 8.53 to 43.16 ± 11.00, a reduction of 8.57 points (*p* < 0.001). On the other hand, the control group had no significant differences in the scores of uremic pruritus (*p* = 0.47), xerostomia (*p* = 0.82), and fatigue (*p* = 0.98) before and after the intervention.

**Conclusion:**

The results of the study demonstrated the impact of auricular acupressure on alleviating pruritus, xerostomia, and fatigue. This complementary medicine technique can be considered as a cost‐effective, safe, acceptable, and noninvasive nursing care option for patients undergoing hemodialysis. Nurses can incorporate auricular acupressure into their practice to provide holistic care for patients undergoing hemodialysis.

**Trial Registration:** Iranian Registry of Clinical Trials: IRCT20151107024919N14

## 1. Introduction

Chronic kidney disease (CKD) is a progressive and irreversible condition characterized by the gradual loss of kidney function over a period of time, ranging from days to years. It is estimated that the global prevalence of CKD is approximately 10%–15% [1, 2]. In 2017, the number of CKD patients was estimated at 752 million people, and 1.2 million of these patients died in the same year [3]. As CKD progresses, it may lead to end‐stage renal disease (ESRD), which necessitates kidney replacement therapy such as dialysis or transplantation to sustain life. Dialysis is employed to remove uremic toxins and maintain electrolyte balance through dietary adjustments [4]. Hemodialysis is the most commonly used form of kidney replacement therapy worldwide, including in Iran [5]. By the end of 2016, the global number of patients undergoing dialysis stood at around 2,989,000, with approximately 89% of them opting for hemodialysis. According to projections, the number of individuals requiring dialysis is expected to reach 4 million by 2020. In Iran, the average prevalence of chronic kidney failure is reported to be 680 individuals per million, which is higher than the global average of 510 individuals per million. Moreover, over 95% of these patients receive hemodialysis treatment [6, 7].

Many patients undergoing intermittent hemodialysis receive treatment at outpatient centers three times a week, with each session lasting 3–4 h [4]. The retention of metabolic waste products and the resulting fluid and electrolyte imbalances have a negative impact on all body systems [8]. Pruritus [9], xerostomia [10, 11], and fatigue [12] are three common problems experienced by these patients.

Pruritus is a prevalent and distressing symptom among individuals with CKD. Uremic pruritus primarily manifests as varying degrees of cutaneous itching, either localized or systemic, commonly affecting the back, limbs, chest, and head. Itching episodes occur intermittently, with varying durations [13]. Patients with milder symptoms may experience brief episodes lasting a few minutes, while those with more severe symptoms may have longer and more pronounced episodes, often exacerbated at night. Epidemiological data suggest that approximately 49% of patients with CKD experience moderate to severe pruritus [14]. The Dialysis Outcomes Practice Patterns Study (DOPPS) conducted from 2012 to 2015 reported that 26–48% of patients experienced at least moderate pruritus, while 13–26% experienced severe or very severe pruritus. Higher degrees of pruritus are associated with increased mortality rates. However, the factors influencing the severity of uremic pruritus in patients are not yet fully understood [15]. Pruritus is common among patients undergoing hemodialysis and is linked to a reduced quality of life and increased mortality. The exact mechanism of pruritus is not fully understood, and treatment options are limited. Accumulation of uremic toxins is believed to be one of the significant factors contributing to pruritus, as evidenced by the relief of symptoms following kidney transplantation [16].

Xerostomia, a commonly overlooked symptom, poses a significant challenge to fluid restriction adherence in patients undergoing hemodialysis. It is estimated that up to 78% of these patients experience xerostomia, making it one of the most prevalent complaints [17]. The reduced saliva flow rate in these individuals is responsible for xerostomia. While the unstimulated salivary flow rate in healthy individuals ranges from 0.3 to 0.5 mL/min, approximately half of patients undergoing hemodialysis have a rate of less than 0.1 mL/min [18]. Xerostomia, although a subjective sensation, can have a negative impact on social activities and personal life. It can lead to difficulties in tasting, chewing, swallowing, and even cause bad breath. Specifically, xerostomia can trigger excessive thirst and increased water intake, leading to noncompliance with fluid restriction regimens. It has been estimated that only 25%–30% of patients undergoing hemodialysis are able to limit their dietary fluid intake to 1 L/day or less [17]. Poor adherence to fluid restriction is associated with interdialytic weight gain (IDWG), which can result in serious complications such as hypertension, congestive heart failure, and even death. Previous approaches to alleviate xerostomia include the use of saliva substitutes like oral pilocarpine and mechanical stimulation of salivary glands through activities like chewing gum. However, saliva substitutes may cause side effects such as flushing, chest tightness, or shortness of breath, while chewing gum may not provide significant relief for xerostomia and can strain the masticatory muscles and teeth. Therefore, it is important to introduce a valid and standardized treatment for controlling xerostomia in patients undergoing hemodialysis [18].

Fatigue is a mental symptom that can range from tiredness to burnout and is associated with various unpleasant feelings such as sickness, weakness, tiredness, and burnout. The continuous lack of energy often leads to stress and frustration, limiting the functional capacity of patients and affecting their physical, cognitive, and social functioning. While fatigue is often viewed from a medical standpoint and attributed to physiological and treatment‐related factors such as renal failure, anemia, inflammation, medication usage, and the severity and frequency of dialysis sessions, an increasing number of studies suggest that fatigue should be understood as a multidimensional phenomenon resulting from the complex interaction of physiological processes, psychosocial phenomena, and behavioral manifestations. Several studies conducted on patients receiving dialysis confirm that, in addition to clinical factors and treatment burden, mood, beliefs, and behaviors also influence the perception of fatigue [12].

Therefore, it is crucial to provide appropriate treatment programs and models to control the disease, reduce complications, and improve the quality of life for patients receiving dialysis. There are various methods available to address these problems, including complementary and alternative medicine, through which nurses can help improve the health of their patients.

Auricular acupressure (AA) is a treatment method derived from traditional Chinese medicine. It involves placing magnetic beads on specific points of the ear and applying appropriate kneading, rubbing, pinching, and pressing techniques to elicit therapeutic effects such as heat, numbness, expansion, and pain. These stimuli are used to prevent and treat diseases and are referred to as AA points. Modern medical research has revealed the presence of numerous nerve tissues in the ear, including the greater auricular nerve, the lesser occipital nerve, branches of the auriculotemporal nerve, the facial nerve, the pharyngeal nerve, the vagus nerve, and the sympathetic nerve, which are connected to the cervical part of the spinal nerve and the cranial nerve, as well as the external carotid artery [19]. In 1990, the World Health Organization (WHO) recognized AA as a treatment for various diseases [20]. Since then, it has been systematically utilized in the management of numerous conditions. Compared to other traditional Chinese medicine techniques, AA is less invasive, more affordable, more acceptable to patients, and easier to implement [21, 22].

Research has shown that xerostomia, pruritus, and fatigue are highly prevalent among patients undergoing hemodialysis and can significantly affect their quality of life. However, to date, no previous study has concurrently evaluated the effect of AA on this triple symptom cluster—pruritus, xerostomia, and fatigue—in hemodialysis patients within a single trial. Furthermore, this is the first clinical trial in southeastern Iran to specifically assess its effect on xerostomia in this population, and it employs a rigorous two‐stage cluster randomization design to prevent contamination between groups—a methodological strength rarely applied in prior acupressure research. These novel aspects provide unique empirical evidence for AA as a safe, cost‐effective, and nurse‐led complementary intervention that addresses multiple distressing symptoms simultaneously. Therefore, this study was conducted to determine the effect of AA on pruritus, xerostomia, and fatigue in patients undergoing hemodialysis in southeastern Iran.

### 1.1. Hypotheses

AA has been found to have a positive effect on pruritus in patients undergoing hemodialysis.

AA has been shown to have a beneficial impact on alleviating xerostomia in patients undergoing hemodialysis.

AA has been found to be effective in reducing fatigue among patients undergoing hemodialysis.

## 2. Materials and Methods

### 2.1. Design

This clinical trial study was conducted among all patients undergoing hemodialysis at the centers affiliated with the Kerman University of Medical Sciences. Kerman University of Medical Sciences, a renowned institution in eastern Iran, is responsible for the supervision of three hemodialysis centers located in Kerman city.

### 2.2. Sampling and Participants

In this study, a significance level of 0.05% and a statistical power of 90% were used.

To determine the sample size for this study, the following formula were used:

(1) n=Z1−α/2+Z1−β2σ12+σ22d2.

According to Cui‐na Yan et al. in China (2015), the SD scores of pruritus were 2.12 (*σ*_1_ = 2.12) and 2.03 (*σ*_2_ = 2.03) in the control and intervention groups, respectively [23]. The study aimed to detect a minimum difference of 1.5 (*d* = 1.5) points between the two groups with a statistical power of 90%. Based on the standard deviation values, the estimated sample size in each group was determined to be 41, resulting in a total of 82 participants [23]. However, to ensure more robust results, 45 participants were included in each group, resulting in a total sample size of 90 patients.

After obtaining the necessary ethical and clinical trial approvals, the researcher proceeded by submitting an introduction letter and visiting the hemodialysis departments of the centers affiliated with the Kerman University of Medical Sciences. Initially, participants who did not meet the inclusion criteria were excluded. Following the selection of eligible patients, demographic and clinical information was collected using a questionnaire specifically designed for patients undergoing hemodialysis.

To prevent contamination between the intervention and control groups (i.e., patients in the control group observing the auricular seeds or receiving information from the intervention group), a two‐stage randomization procedure was used.

First stage (cluster formation based on dialysis days): patients dialyzing on odd‐numbered days (Saturday, Monday, Wednesday) were assigned to one cluster, and those dialyzing on even‐numbered days (Sunday, Tuesday, Thursday) were assigned to another cluster. These two clusters were physically separated in time and space within the dialysis units.

Second stage (random allocation of clusters): after the two clusters were formed, a computer‐generated simple randomization sequence (1:1 ratio) was used to randomly assign one cluster to the intervention group and the other to the control group. The randomization sequence was prepared by a statistician not involved in the intervention, and allocation was concealed until the assignment was made.

After the two‐stage randomization was completed, the intervention phase commenced. Participants were unaware of their group allocation. The statistician responsible for data analysis and the outcome assessors remained blinded. However, blinding was not implemented by the researcher who administered the intervention (care provider), as group allocation was evident based on dialysis days. Vaccaria seeds from the same brand (Zhongyan Taihe company) were used to maintain consistency.

A total of 90 dialysis patients experiencing symptoms of pruritus, fatigue, and xerostomia were identified based on self‐completed questionnaires. From this pool of eligible patients, 45 individuals were selected for the intervention group and 45 individuals for the control group. Patients were instructed to report any changes in their diet or medication. Additionally, they were trained on the proper storage and usage of Vaccaria seeds.

Inclusion criteria for this study consisted of individuals aged 18–75 years [24] who underwent regular hemodialysis (two or three sessions per week) [18]. Additionally, participants were required to have a minimum score of 3 points on the VAS for pruritus [23], as well as experience symptoms of xerostomia [21] and fatigue in the previous month.

Exclusion criteria encompassed the presence of concomitant diseases such as cancer, congestive heart failure, connective tissue disease, hematological diseases, and hepatic diseases [17, 18, 23]. Individuals with severe physical symptoms that clearly caused insomnia and fatigue, such as bone pain, sleep apnea, and restless legs, were also excluded [22]. Fatigue caused by severe anemia (hemoglobin < 60 g/L) or malnutrition (serum albumin < 30 g/L) was considered an exclusion criterion as well [25]. Other exclusion criteria included ear infections [18, 23], allergy to Vaccaria seeds [17, 18], use of medications that might cause xerostomia (such as anticholinergics, tricyclic antidepressants, and beta‐blockers) [18, 21], and the use of drugs that might cause pruritus (such as drugs and substances to which the patient is allergic, narcotics, antifungals).

During the course of the study, one patient discontinued the intervention due to migration to another city, and two patients chose not to continue with the AA procedure. The three dropouts were replaced with new eligible patients who received the same allocation and completed the 4‐week protocol. The final analysis included all 90 enrolled patients.

To ensure adherence, the researcher was present at the centers on hemodialysis days to directly monitor the placement of seeds and the acupressure technique. On nondialysis days, patients recorded their self‐acupressure sessions in a daily diary and received telephone follow‐up from the researcher. The diary was reviewed at each dialysis session. No patient was excluded due to poor adherence, and all patients demonstrated acceptable compliance with the self‐acupressure protocol based on the researcher’s monitoring and diary reviews.

### 2.3. Data Collection

The questionnaires used in this study are as follows: a demographic and background information form, the Summated Xerostomia Inventory (SXI), the Visual Analog Scale (VAS), and the Fatigue Severity Scale (FSS).

All of the aforementioned questionnaires were administered to patients undergoing hemodialysis in the intervention and control groups both before the intervention and 4 weeks after the intervention. The completed questionnaires were collected from the participants for further analysis.

The demographic and background information form included age, sex, education level, marital status, illness diagnosis, number of hemodialysis sessions, hypertension, interdialytic weight, pruritus score, presence or absence of fatigue, and xerostomia in the past month.

The SXI was developed by Thomson et al. to assess xerostomia. This inventory consists of five items, each rated on a 5‐point scale ranging from “*never*” to “*very much*” [26]. In a translated version conducted by Hosseini et al. [27], the internal consistency was evaluated using Cronbach’s alpha coefficient, while the external reliability was assessed through the test–retest method with a 2‐week interval, using the intraclass correlation coefficient. Construct validity was examined using confirmatory factor analysis and fit indices.

Following the face and content validity of the Persian version of the questionnaire, no changes were made to the items. The internal reliability of the questionnaire was determined to be 0.81, as measured by Cronbach’s alpha coefficient. The test–retest analysis showed no significant difference in mean scores, with an intraclass correlation coefficient of 0.67. Construct validity was assessed using confirmatory factor analysis on the 6‐item version, where all items demonstrated factor loadings higher than 0.04. These findings indicated that the questionnaire possessed satisfactory validity and reliability within the Iranian population, making it a suitable and straightforward tool for measuring xerostomia [27].

The VAS was initially introduced by Hayes [28]. The VAS is widely used as one of the most common methods due to its simplicity and efficiency in estimating pruritus intensity. The VAS consists of a 10‐cm long line, which can be either horizontal or vertical. Patients mark the intensity of their itching that corresponds to the severity of experienced itching, with 0 indicating the absence of itching and 10 representing the most severe itching [29, 30].

The FSS, developed by Krupp et al. [31], measures an individual’s personal perception of fatigue. It consists of nine items, with each item rated on a seven‐point scale. Patients with a mean score of 4 or higher are considered to experience significant fatigue [31, 32]. This scale is commonly employed in medical research.

The validity and reliability of the Persian version of the FSS have been examined by Shahvarughi Farahani et al. A Cronbach’s alpha coefficient of 0.96 indicated high internal consistency when assessing the questionnaire, and the items of the scale measured the same underlying concept. The correlation between each item and the other items was higher than the desired level, even after accounting for any potential overlap. The test–retest reliability of the Persian version of the scale was assessed with a correlation coefficient of 0.93, which surpasses the acceptable value of 0.7. These findings demonstrate that the FSS exhibits good reliability and validity in the Persian context [33].

Primary outcomes were defined as the mean changes in pruritus (measured by VAS) and xerostomia (measured by SXI) scores from baseline to 4 weeks. Secondary outcome was defined as the mean change in fatigue (measured by FSS) scores over the same period.

### 2.4. Intervention

After obtaining the necessary ethical approval from the Ethics Committee of the Kerman University of Medical Sciences and the clinical trial code from the Iranian Registry of Clinical Trials, the researcher visited the hemodialysis departments of the centers affiliated with the Kerman University of Medical Sciences, presenting a letter of introduction. Eligible patients were identified, and their demographic and clinical characteristics were recorded. The intervention was then implemented based on the group allocation previously determined by the two‐stage randomization procedure.

The AA points were identified and coded according to the Chinese National Standard GB/T 13734‐2008 “Nomenclature and location of auricular points” [34]. The points used in this study included TF4 (Shenmen), AH6a (Sympathetic), CO10 (Kidney), CO13 (Spleen), CO18 (Endocrine), TG1 (Upper Tragus), CO1 (Mouth), and AT4 (Subcortex) [35].

After identifying the desired points, the patient’s ear was cleaned with a 70% alcohol disinfectant. The Vaccaria seeds, obtained from Zhongyan Taihe Company, were then attached using medical tapes to one ear of each patient (unilateral application).

The seeds were placed around the patient’s ear, and the researcher applied pressure until the patient reported the characteristic “Deqi” sensation (including feelings of soreness, numbness, distension, or warmth). The pressure was then maintained for approximately 30–60 s, adjusting the duration according to each patient’s individual sensitivity to achieve and sustain the Deqi sensation. The pressing force was estimated to be approximately 3–5 Newtons (N), which corresponds to a comfortable but perceptible pressure without causing pain or tissue damage.

Patients were instructed to perform self‐acupressure three times daily (morning, noon, and evening) and additionally whenever they felt xerostomia. For each self‐massage session, they were asked to press each seed until a mild Deqi sensation was perceived (soreness, numbness, or distension) and to maintain the pressure for 10–15 s per point, repeating the pressing movement 10–15 times per session.

To prevent excessive stimulation and skin irritation, the seeds were alternated between the right and left ears at each dressing change. Specifically, the seeds were replaced every 2‐3 days, coinciding with the patients’ hemodialysis sessions. At each session, the old seeds and tape were removed from one ear, and new seeds were applied to the opposite ear.

The control group received routine hemodialysis care, including monitoring of vital signs, weight, fluid intake, medication administration as prescribed, and standard nursing education on diet and fluid restriction, without any AA intervention.

The intervention was conducted under the supervision of a traditional medicine expert (fourth author). Participants were instructed not to change their medications or diet during the intervention to control for confounding factors. They were also advised to avoid taking drugs or substances that cause pruritus, xerostomia, or increased saliva secretion (e.g., donepezil, salbutamol, diuretics, Kin Hidrat, Cevimeline) and to inform the researcher before starting any new medication.

No adverse events, including skin allergy, local pain, auricular infection, or tape‐related dermatitis, were reported in either group during the 4‐week intervention period. Patients were instructed to report any discomfort immediately, and no such reports were received.

### 2.5. Analysis

After the data were collected, they were imported into SPSS26 for analysis. Descriptive statistics such as prevalence, percentage, mean, and standard deviation were used to describe the data. To compare demographic variables such as age and sex, independent t‐ and chi‐squared tests were employed. Paired *t*‐test was conducted to compare the scores of pruritus, xerostomia, and fatigue within each group at the beginning and end of the study. To control for potential confounding variables, analysis of covariance (ANCOVA) was used. Baseline (preintervention) scores were selected as the primary covariate due to their theoretical and statistical relevance in reducing residual variance, as recommended for pretest–posttest designs. Other baseline characteristics, including age, sex, marital status, education level, and occupation, were initially considered as covariates. However, because the two groups were successfully matched on these variables at baseline (Table 1), they were excluded from the model to maintain parsimony. A significance level of 0.05 was considered statistically significant.

**TABLE 1:** Comparison of participants’ demographic characteristics.

Variable		Experimental group	Control group	*χ* ^2^/*t*	*p* value
		*n* (%)/M ± SD	*n* (%)/M ± SD		
Age		53.82 ± 11.73	52.13 ± 12.20	−0.66	0.51

Sex	Male	24 (53)	27 (60)	0.4	0.52
	Female	21 (46.7)	18 (40)		

Marital status	Married	35 (77.8)	36 (80)	0.06	0.79
	Single	10 (22.2)	9 (20)		

Occupation	Self‐employed	9 (20)	10 (22.2)	0.44	0.93
	Employed	8 (17.8)	10 (22.2)		
	Housewife	20 (44.4)	18 (40)		
	Unemployed	8 (17.8)	7 (15.6)		

Education level	Lower/upper secondary education	19 (42.2)	18 (40)	3.44	0.32
	Diploma	17 (38.8)	11 (24.4)		
	Bachelor’s degree	8 (17.8)	15 (33.3)		
	Master’s degree	1 (2.2)	1 (2.2)		

### 2.6. Ethical Considerations

The Ethics Committee of the Kerman University of Medical Sciences approved this project under the ID No. IR.KMU.REC.1401.316 on 7.11.2022.

Prior to their participation in the study, all participants provided written informed consent. They were informed about their right to withdraw from the study at any point, without any impact on their ongoing care. Participants were also made aware that any side effects, such as local itching, could be promptly alleviated by removing the medical tape.

Confidentiality was guaranteed for all participants, and the research adhered to the principles outlined in the Declaration of Helsinki and the Committee on Publication Ethics (COPE).

## 3. Results

The study findings revealed that the mean age of participants in the intervention group was 53.82 ± 11.73 years, while it was 52.13 ± 12.20 years in the control group. The independent *t*‐test indicated that there was no significant difference in age between the two groups (*p* < 0.05).

The majority of participants were women (46.7% in the intervention group and 40% in the control group). Furthermore, most of the participants were married (77.8% in the intervention group and 80% in the control group). The majority of participants were housewives (44.4% in the intervention group and 40% in the control group), and the highest level of education among participants was lower/upper secondary (42.2% in the intervention group and 40% in the control group). For educational level, the chi‐squared test (*χ* ^2^ = 3.44, *p* = 0.32) suggests no significant difference between the two groups. Although the proportion of participants with a bachelor’s degree appeared to be numerically different between the two groups (17.8% vs. 33.3%), the chi‐squared test assesses the overall distribution across all educational categories, rather than comparing a single category independently. When all educational categories are considered simultaneously, the overall distribution between the two groups is not significantly different. As shown in Table 1, there were no significant differences between the intervention and control groups in terms of age, sex, marital status, occupation, and educational level (all *p* > 0.05), indicating successful matching of the two groups at baseline (Table 1).

### 3.1. Pruritus

The results pertaining to the itching condition of the participants indicated that the mean pruritus scores in the control and intervention groups were 5.98 (±2.43) and 6.11 (±2.32), respectively, before the intervention. The independent *t*‐test revealed no significant difference in pruritus scores between the two groups before the intervention (*p* = 0.79) (Table 2).

**TABLE 2:** Comparison of pruritus, fatigue, and xerostomia between intervention and control groups before the intervention.

		Mean	SD	Mean difference	C.I 95%	*T*	*p* value
Pruritus	Control	5.98	2.43	−0.13	−1.12, 0.86	−0.26	0.79
	Intervention	6.11	2.32				

Fatigue	Control	49.29	7.44	−2.44	−5.79, 0.91	−1.44	0.15
	Intervention	51.73	8.53				

Xerostomia	Control	18.56	2.32	−1.08	−2.61, 0.43	−1.42	0.15
	Intervention	19.64	3.78				

There was a significant difference in the pruritus scores of the intervention group before and after the intervention. The mean pruritus score decreased from 6.11 to 3.18, indicating a significant reduction of 2.93 points (*p* < 0.001). There was no significant difference in pruritus scores of the control group before and after the intervention. The mean pruritus score decreased from 5.98 to 5.84, with a minimal reduction of 0.13 points (*p* = 0.47) (Table 3).

**TABLE 3:** Comparison of pruritus, fatigue, and xerostomia between intervention and control groups before and after the intervention.

		Mean (SD) before the intervention	Mean (SD) after the intervention	Mean difference (C.I 95%)	*T*	*p* value
Pruritus	Control	5.98 ± 2.42	5.84 ± 2.59	0.13 (−0.24, 0.51)	0.71	0.47
	Intervention	6.11 ± 2.32	3.18 ± 2.74	2.93 (2.34, 3.5)	10.10	< 0.001

Xerostomia	Control	18.56 ± 3.78	18.64 ± 3.92	−0.08 (−0.91, 0.73)	−0.21	0.82
	Intervention	19.64 ± 3.46	15.62 ± 5.29	4.02 (2.61, 5.43)	5.75	< 0.001

Fatigue	Control	49.29 ± 7.44	49.31 ± 7.24	−0.02 (−1.85, 1.81)	−0.02	0.98
	Intervention	51.73 ± 8.53	43.16 ± 11.00	8.57 (5.8, 11.34)	11.34	< 0.001

The results of the ANCOVA, with baseline scores as the covariate, demonstrated a significant difference in the pruritus scores between the two groups after the intervention. Specifically, the pruritus score in the intervention group was 2.78 points lower than that of the control group after the intervention (*p* < 0.001) (Table 4).

**TABLE 4:** Determining the mean scores of pruritus, fatigue, and xerostomia in two groups before and after the intervention using the ANCOVA test.

		Mean	SD	Mean difference	C.I 95%	*F*	*p* value^∗^
Pruritus	Control	5.98	2.43	2.78	2.1, 3.46	65.9	< 0.001
	Intervention	3.18	2.32				

Fatigue	Control	49.29	7.44	7.86	4.69, 11.04	24.26	< 0.001
	Intervention	43.16	8.53				

Xerostomia	Control	18.64	2.32	3.85	2.25, 5.45	23.03	< 0.001
	Intervention	15.62	3.78				

^∗^Adjust for before.

### 3.2. Fatigue

The results indicated that the mean fatigue scores of the control and intervention groups were 49.29 (±7.44) and 51.73 (±8.53), respectively, before the intervention. The independent *t*‐test reported no significant difference in fatigue between the two groups before the intervention (*p* = 0.15) (Table 2).

The intervention group showed a significant reduction in fatigue scores, from a mean of 51.73 to 43.16 (a decrease of 8.57 points, *p* < 0.001). In contrast, the control group exhibited no significant change, with scores shifting minimally from 49.29 to 49.31 (difference of 0.02, *p* = 0.98) (Table 3). Although the standard deviation in the intervention group increased from 8.53 to 11.00 after the intervention, this reflects greater variability in individual responses to acupressure rather than measurement error, as the same validated instrument (the FSS) was used under identical conditions. Furthermore, ANCOVA analysis adjusted for baseline fatigue scores confirmed a significant between‐group difference postintervention, with the intervention group scoring 7.86 points lower than the control group (*p* < 0.001) (Table 4). These findings indicate that the treatment effect remains robust despite increased interindividual variability in responses.

### 3.3. Xerostomia

The results indicated that the mean xerostomia score in the control group was 18.56 (±2.32) before the intervention, while it was 19.64 (±3.78) in the intervention group. The independent *t*‐test indicated no significant difference in the xerostomia scores of the two groups before the intervention (*p* = 0.15) (Table 2).

A significant difference was observed in the xerostomia scores of the intervention group before and after the intervention. The mean xerostomia score decreased from 19.64 to 15.62, indicating a significant reduction of 4.02 points (*p* < 0.001). Conversely, there was no significant difference in xerostomia scores of the control group before and after the intervention. The mean xerostomia score changed minimally from 18.56 to 18.64, with a difference of 0.08 points, which was not statistically significant (*p* = 0.82) (Table 3).

The results of the ANCOVA, with baseline scores as the covariate, demonstrated a significant difference in the xerostomia scores between the two groups after the intervention. Specifically, the xerostomia score in the intervention group was 3.85 points lower than that of the control group after the intervention (*p* < 0.001) (Table 4).

## 4. Discussion

The current study aimed to investigate the impact of AA on pruritus, xerostomia, and fatigue in patients undergoing hemodialysis. The findings revealed a significant reduction in pruritus in the intervention group compared to that in the control group. This aligns with the study conducted by Cui‐na Yan et al., where they observed a decrease in uremic pruritus and serum histamine levels in the intervention group following AA during a 6‐week follow‐up period. Their findings suggested that AA could alleviate itching symptoms in patients undergoing hemodialysis by reducing histamine levels [23]. Similarly, a review study by Panma et al. indicated that acupressure might hold promise as a treatment option for pruritus in patients undergoing hemodialysis, although further research is needed to establish its effectiveness [36]. The present study supports previous research that AA can effectively reduce pruritus in patients undergoing hemodialysis.

Mast cells are crucial immune cells that have been extensively studied in relation to uremic pruritus in patients with ESRD [13, 37]. Research has demonstrated an increased number of mast cells in the skin of ESRD patients experiencing uremic pruritus [37]. These mast cells play a significant role in the pathogenesis of pruritus, as they release histamine, which acts as the primary mediator, affecting the keratinocytes, the main component of the skin [38]. Mast cells are capable of releasing various substances, including histamine, tumor necrosis factor (TNF), and interleukins, which are commonly recognized as inflammatory markers [39]. Moreover, mast cells are essential immune cells involved in diverse biological reactions, immune defense mechanisms, endocrine regulation, and axonal reflexes. In addition to their role in hypersensitivity reactions, mast cells also contribute to various immune functions, encompassing both innate and adaptive immunity [38].

The findings of the current study indicated that AA could reduce xerostomia in the intervention group compared to that in the control group. Chang et al. demonstrated that the combined use of AA and a fluid restriction program had positive effects on improving the salivary flow rate, body fluid control, interdialytic weight, and quality of life related to dietary changes in patients undergoing hemodialysis. This approach was deemed cost‐effective, safe, and complementary for these patients [18]. Similarly, Jung and Chang found that AA reduced xerostomia and constipation, improved saliva flow rate, and enhanced diet‐related quality of life. The noninvasive nature of AA made it a recommended effective and independent nursing intervention [21]. Additionally, Yang et al. reported a significant decrease in xerostomia in patients undergoing hemodialysis after a 4‐week intervention [24]. The present study’s results align with these previous studies, further supporting the effectiveness of AA in reducing xerostomia in patients undergoing hemodialysis.

Xerostomia is a prevalent symptom among patients undergoing hemodialysis and can have a significant impact on their quality of life. The exact cause of xerostomia in these patients is often attributed to the atrophy and fibrosis of salivary glands, although the underlying origin remains unknown [40]. Furthermore, many medications commonly prescribed to dialysis patients can contribute to or worsen xerostomia symptoms. The consequences of xerostomia can be distressing and have a profound effect on various aspects of daily life, including difficulties in chewing, swallowing, tasting, and speaking. Additionally, xerostomia can lead to an increased risk of oral diseases such as mucosal, gum, and tongue lesions, as well as bacterial and fungal infections like candidiasis. It can also contribute to tooth decay, periodontal disease, interdialytic weight due to increased fluid consumption, and a reduced overall quality of life [41].

The findings of the present study indicated a significant reduction in the mean fatigue score of the intervention group compared to that of the control group. Bossola demonstrated that AA, as a simple and noninvasive method, was effective in reducing pruritus and fatigue and alleviating symptoms reported by patients undergoing hemodialysis [40]. Similarly, Huang highlighted the importance of evaluating the energy levels of chakras in patients experiencing chronic fatigue after hemodialysis sessions. He suggested that compensating for and increasing the energy of chakras through AA could be a valuable approach in treating fatigue and preventing future fatigue episodes associated with hemodialysis sessions [42]. The results of the present study align with these previous findings, further supporting the effectiveness of AA in reducing fatigue in patients undergoing hemodialysis.

The present study used AA treatment, a traditional Chinese medicine approach that shares the same purpose as acupuncture. This method is known for its simplicity, convenience, and affordability, making it widely used in clinical disease management. Although the exact mechanism of AA remains unknown, it is believed to activate energy channels, regulate and balance energy flow in the body, and have positive effects on hormone levels, neurotransmitter release (such as serotonin), blood circulation, relaxation, brain stimulation, and immune system enhancement [43]. Recent evidence suggests that auriculotherapy can be explained more by its neuroanatomical and neurophysiological basis than by the traditional concepts of energy channels. The earlobe is extensively innervated by the auricular branch of the vagus nerve, the greater auricular nerve, the temporal auricular nerve, the facial nerve, and the glossopharyngeal nerve. Stimulation of these neural pathways may modulate the activity of the autonomic nervous system, brainstem nuclei, central pain processing networks, and inflammatory pathways, thereby helping to improve itching, dry mouth, and fatigue [19, 44, 45]. AA can produce a calming and analgesic effect by stimulating the subcortical point and by modulating the excitement of the autonomic nervous center and the cortical and subcortical inhibition processes [46]. It is important to note that the practical application of research results is crucial for improving the current situation and addressing societal issues. Therefore, the findings of this study can be used in nursing management, education, and research. Also, it is very important to consult with patients and their families about using the results of this research to improve their symptoms. Counseling plays a significant role in managing chronic diseases and provides patients with guidance to recognize and explore their issues.

### 4.1. Limitation

There were certain limitations to this study. Firstly, the sample size was small, and the follow‐up period was short. Future research with a larger sample size, a control group, and a longer follow‐up duration is recommended. Additionally, since the data collection was limited to a 4‐week period after the treatment, there is a lack of information about the long‐term effects of AA. It is suggested that patients be re‐evaluated for pruritus, xerostomia, and fatigue symptoms 1 month after the intervention. Lastly, it is important to acknowledge that the study participants were specific to the Kerman population; therefore, the generalizability of the findings to other populations may be limited.

## 5. Conclusion

The results of the present study demonstrated a statistically significant difference in the mean scores of pruritus, xerostomia, and fatigue between the intervention and control groups, with a significant decrease observed in the intervention group. Therefore, AA can be considered as a viable treatment option to alleviate these symptoms in patients undergoing hemodialysis.

## Author Contributions

Batool Tirgari, Ehsan Behjat Jabari, and Mohammad Setayesh designed the study. Batool Tirgari and Mansooreh Azizzadeh Forouzi trained the intervention method. Ehsan Behjat Jabari collected data. Yunes Jahani analyzed data and prepared tables and results. Ehsan Behjat Jabari, Batool Tirgari, Mansooreh Azizzadeh Forouzi, Tori Canillas Dufau, and Mohammad Setayesh interpreted the data. All authors made a significant contribution to writing the manuscript.

## Funding

There is no funding support.

## Disclosure

All authors have read and approved the final manuscript.

## Ethics Statement

This project was approved by the Ethic Committee of the Kerman University of Medical Sciences (IR.KMU.REC.1401.316). All the participants signed a written informed consent before their inclusion in this project, and the participants were informed that they could withdraw from the study at any time, without affecting their care and treatment. There was no ethical issue during the study and data collection. Moreover, they were ensured about confidentiality of information, and all the data were analyzed anonymously, and clinical investigation was conducted according to the principles expressed in the Declaration of Helsinki and Committee on Publication Ethics (COPE).

## Consent

Please see the Ethics Statement.

## Conflicts of Interest

The authors declare no conflicts of interest.

## Data Availability

The authors make all datasets available to editors and reviewers; for the datasets, contact the corresponding author.
